# Effects of a new speech support application on intensive speech therapy and changes in functional brain connectivity in patients with post-stroke aphasia

**DOI:** 10.3389/fnhum.2022.870733

**Published:** 2022-09-22

**Authors:** Yuta Katsuno, Yoshino Ueki, Keiichi Ito, Satona Murakami, Kiminori Aoyama, Naoya Oishi, Hirohito Kan, Noriyuki Matsukawa, Katashi Nagao, Hiroshi Tatsumi

**Affiliations:** ^1^Department of Rehabilitation Medicine, Japanese Red Cross Aichi Medical Center Nagoya Daini Hospital, Nagoya, Japan; ^2^Department of Rehabilitation Medicine, Nagoya City University Graduate School of Medical Sciences, Nagoya, Japan; ^3^Department of Rehabilitation Medicine, Kamiida Rehabilitation Hospital, Nagoya, Japan; ^4^Medical Innovation Centre, Kyoto University Graduate School of Medicine, Kyoto, Japan; ^5^Department of Integrated Health Sciences, Nagoya University Graduate School of Medicine, Nagoya, Japan; ^6^Department of Neurology, Nagoya City University Graduate School of Medical Sciences, Nagoya, Japan; ^7^Department of Intelligent Systems, Graduate School of Informatics, Nagoya University, Nagoya, Japan; ^8^Department of Health Science, Aichi Gakuin University, Nagoya, Japan

**Keywords:** aphasia, functional connectivity, language network, resting-state functional magnetic resonance imaging, speech support application

## Abstract

Aphasia is a language disorder that occurs after a stroke and impairs listening, speaking, reading, writing, and calculation skills. Patients with post-stroke aphasia in Japan are increasing due to population aging and the advancement of medical treatment. Opportunities for adequate speech therapy in chronic stroke are limited due to time constraints. Recent studies have reported that intensive speech therapy for a short period of time or continuous speech therapy using high-tech equipment, including speech applications (apps, can improve aphasia even in the chronic stage. However, its underlying mechanism for improving language function and its effect on other cognitive functions remains unclear. In the present study, we investigated whether intensive speech therapy using a newly developed speech support app could improve aphasia and other cognitive functions in patients with chronic stroke. Furthermore, we examined whether it can alter the brain network related to language and other cortical areas. Thus, we conducted a prospective, single-comparison study to examine the effects of a new speech support app on language and cognitive functions and used resting state functional MRI (rs-fMRI) regions of interest (ROI) to ROI analysis to determine changes in the related brain network. Two patients with chronic stroke participated in this study. They used the independent speech therapy system to perform eight sets of 20 randomly presented words/time (taking approximately 20 min), for 8 consecutive weeks. Their language, higher cognitive functions including attention function, and rs-fMRI, were evaluated before and after the rehabilitation intervention using the speech support app. Both patients had improved pronunciation, daily conversational situations, and attention. The rs-fMRI analysis showed increased functional connectivity of brain regions associated with language and attention related areas. Our results show that intensive speech therapy using this speech support app can improve language and attention functions even in the chronic stage of stroke, and may be a useful tool for patients with aphasia. In the future, we will conduct longitudinal studies with larger numbers of patients, which we hope will continue the trends seen in the current study, and provide even stronger evidence for the usefulness of this new speech support app.

## Introduction

The number of stroke victims in Japan is estimated to be 106,506 per year (Ministry of Health, Labor and Welfare in Japan, 2019), and it is the fourth leading cause of death. The number of patients with aphasia after stroke is increasing, due to an aging of society and the availability of advanced medical treatment not only in Japan but also in the world (Engelter et al., 2006; Dickey et al., 2010; National Institute on Deafness and Other Communication Disorders, 2017). Many studies have shown the benefit of rehabilitation in the acute (Laska et al., 2011; Godecke et al., 2012) and chronic (Allen et al., 2012) phases after stroke. These studies underscore the importance of providing sustained rehabilitation to patients in acute and chronic settings.

Aphasia is a language disorder that impairs any or all language functions, including listening, speaking, reading, writing, and calculating, and is a cause of decreased quality of life. On the other hand, opportunities for adequate speech and language therapy after the chronic phase are limited in Japan, because of the shortage of specialized therapists and the time constraints of rehabilitation. However, it has been reported that short-term intensive speech therapy contributes to the improvement of language function (Code et al., 2010). Classical speech therapy includes general individual speech therapy, group training, gesture cues, and constraint-induced movement therapy. However, most are generally performed by a speech therapist face to face with the patient. In Japan, there is a cap on the number of therapy sessions; therefore, patients whose aphasia remains until the chronic phase may not receive adequate speech therapy. New rehabilitation methods are increasingly incorporating high-tech equipment such as computers and tablet devices for speech therapy. A systematic review by Des Roches and Kiran (2017) on aphasia rehabilitation using new technologies reported that speech therapy methods vary across studies and ultimately show consistent improvements in various outcome measures.

Several studies have reported about the effectiveness of rehabilitation using computer programs in patients with aphasia. Pedersen et al. (2001) reported that all participants improved their naming after computer therapy using a program that incorporated semantic, phonological, and other cues. Palmer et al. (2012) also conducted a randomized controlled trial of computer therapy in patients with chronic aphasia. They found that after 5 months of computer therapy, the computer therapy group showed significant improvement in naming function compared to the control group.

Improvements in speech fluency and naming function have been observed after using tablet-based speech rehabilitation in patients with chronic aphasia (Fridriksson et al., 2012, 2015; Cassarly et al., 2021). Patients with aphasia who participated in another tablet-based home therapy program were able to widen their vocabulary during the intensive phase of the therapy and learn new words with daily practice for an additional 6 months (Kurland et al., 2014, 2018). These showed that speech therapy using tablet devices can help even patients with chronic severe aphasia to improve their language function.

These studies offer promising prospects for the future of speech therapy using high-tech equipment including computers and tablets. However, its underlying mechanism for improving language function and its effect on other cognitive functions remains unclear. In the present study, we investigated whether intensive speech therapy using a newly developed speech support application could improve aphasia and other cognitive functions in patients with chronic stroke. Furthermore, we examined whether it can alter the brain network related to language and other cortical areas.

## Materials and methods

**Figure 1:**
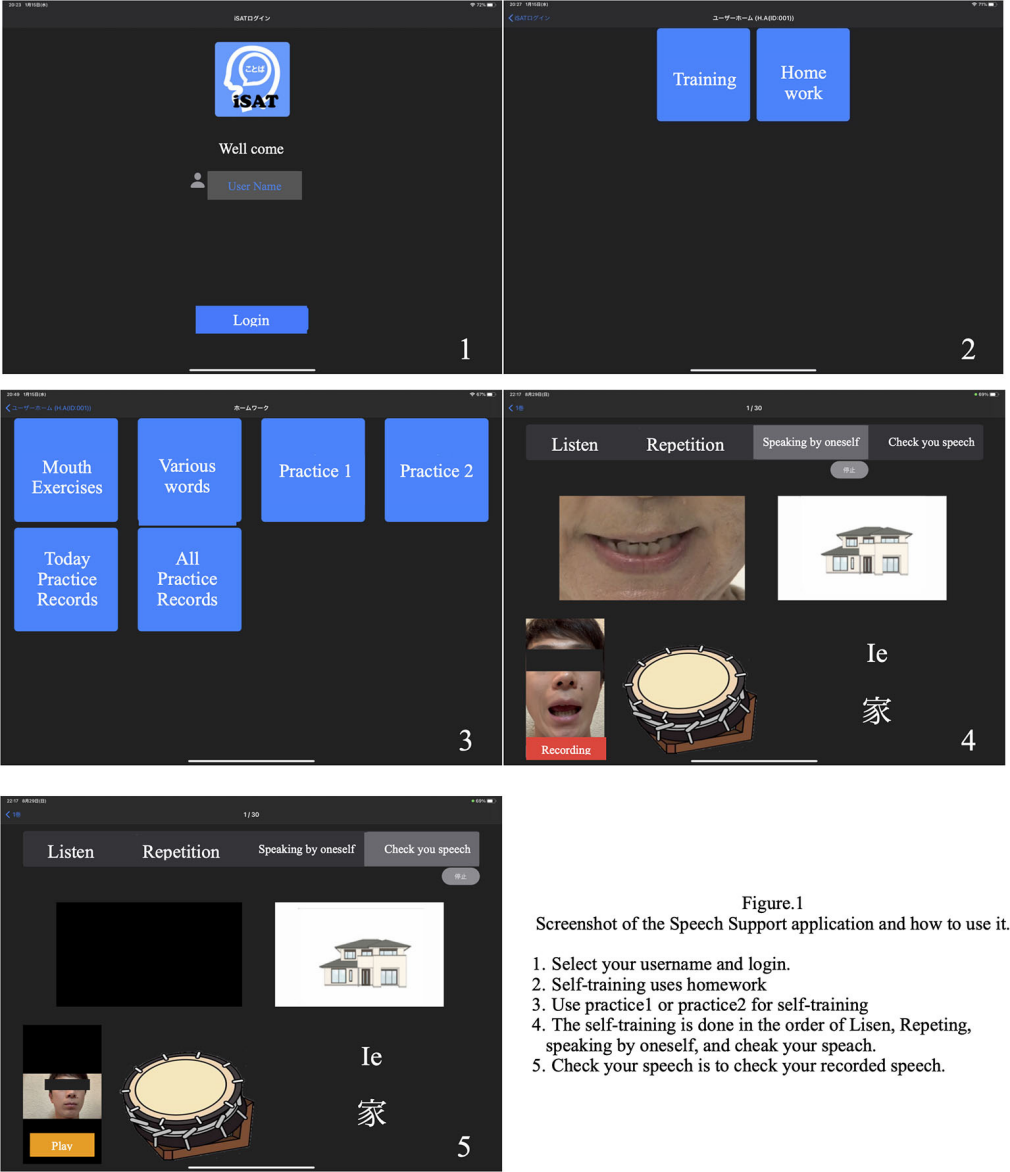
Speech therapy support application screen.

We developed a speech therapy support tool, the “Speech Support App.” The Speech Support App (Figure 1) is used on an iPad Pro and has the following functions: (1) speech exercises; (2) everyday phrases (about 200 words); (3) words (1,200 nouns, 300 verbs); and (4) short sentences (more than 700 sentences). The speech support application screen shows repeatedly and simultaneously an oral model of speech (where the speech rate can be adjusted on each trial), picture cards, and text stimuli; and the data of each trial are simultaneously recorded. Since the patients' speech practices are recorded on the iPad, they can check it by themselves. Moreover, a calendar function is provided, allowing patients to check the number of their trials in each day. The speech therapist can review video recordings of the patient's independent speech training and can provide feedback to the patient on the status of their practice. The application is also equipped with an automatic recording function, and it is possible to record the number of times the patient has conducted independent speech therapy. In addition, if a word is not included in the list, the user can create it themselves.

### Subjects

In this study, two patients presented with aphasia after stroke: a man in his 50 s and a woman in her 60 s, both with left putamen hemorrhage and similar language difficulties. More than 2 years had passed since the onset of the disease in both patients. Although they retained auditory and reading comprehension, they presented serious residual apraxia of speech and naming difficulty, showing motor aphasia (Broca's aphasia). They were highly motivated for speech therapy; however, they presented complications such as fatigue and poor concentration when receiving face-to-face speech therapy. Therefore, we recommended the use of the Speech Support App to improve their apraxia of speech.

This study was planned and carried out in accordance with the ethical regulations of the Ethics Committee of Kamiida Rehabilitation Hospital. The use of the Speech Support App and the purpose of the study were explained using a consent form, and written consent was obtained from the participants and their families.

### Method and duration

**Figure 2:**
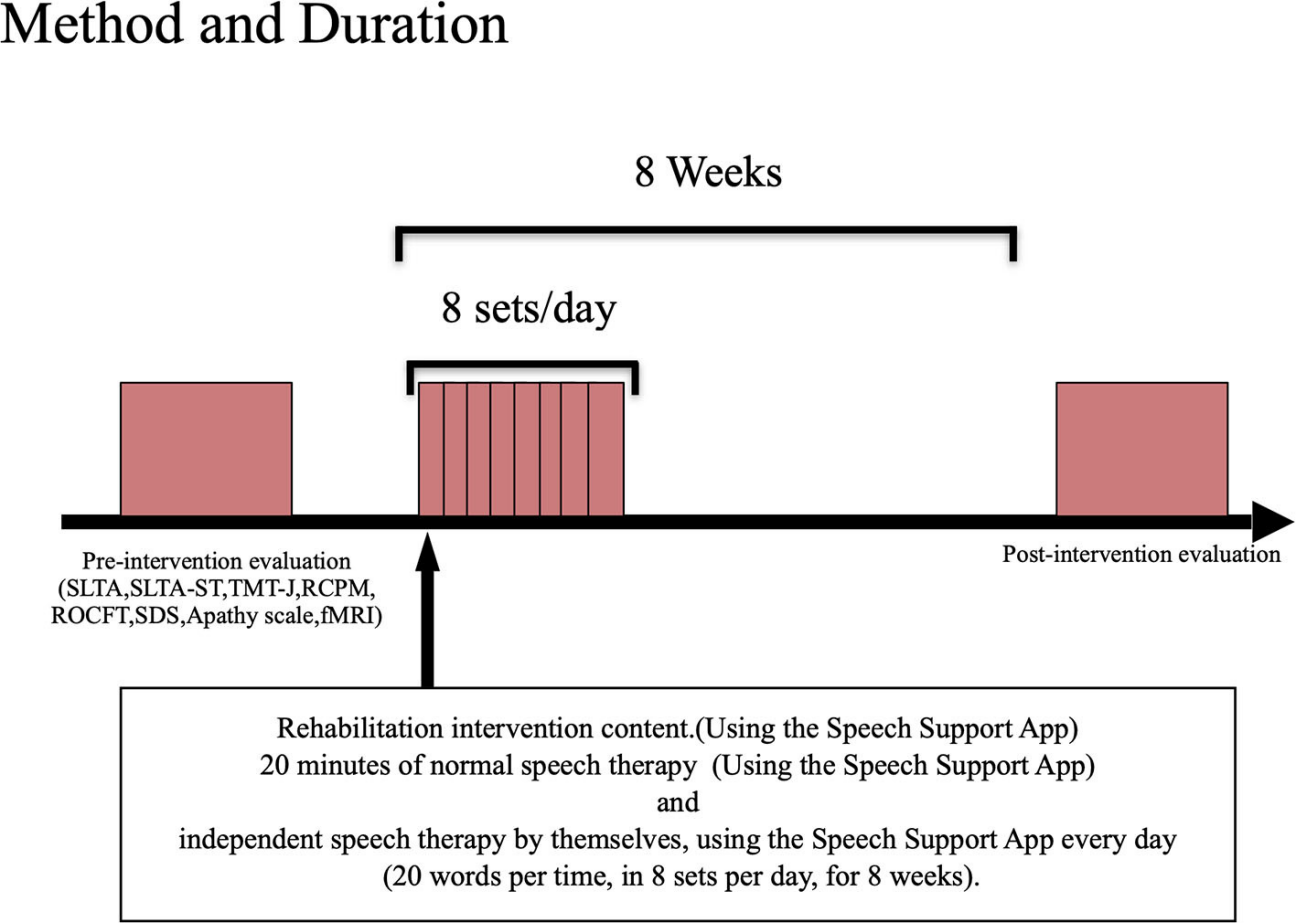
Evaluation/intervention flow.

The participants were tested before and after the intervention using the Speech Support App (Figure 2). The Speech Support App was used for 20 min of speech therapy provided by the speech therapist. In addition, the patients were instructed to perform intensive independent speech therapy by themselves, using the Speech Support App every day (20 words per set, in 8 sets per day, for 8 weeks). The speech therapist in charge of the patient checked the implementation record on the iPad to ensure that the patient was using the Speech Support App. The same evaluations (language function, higher cognitive functions, attention function, imaging, and other evaluations) were conducted before and after the rehabilitation intervention. The current study also examined the secondary effects of longer periods (8 weeks) of independent speech therapy with the speech support application to see if improvements in higher cognitive functions other than language function could be obtained.

### Rehabilitation interventions

Using the Speech Support App installed on the iPad, the patients were trained to say words and short sentences using complex stimuli (oral images + voice + letters + picture cards). The patients performed speech therapy in the following order: recitation, recapitulation, and self-utterance. Intensive speech therapy was performed in an inpatient therapy program using the Speech Support App. Additionally, the patient independently performed speech therapy using the Speech Support App outside of the speech therapy program, by using the language tasks in the App selected by the speech therapist. These language tasks were set at the task level by the speech therapist in charge and were randomly displayed from among the set tasks during independent speech therapy. The speech therapist in charge later confirmed the implementation status.

### Methods for evaluating language and higher cognitive functions

To evaluate the effect of using the Speech Support App, we performed the same language function, higher cognitive functions, attention function, imaging, and other evaluations before and after the rehabilitation intervention.

To provide individual patient with intensive speech therapy using a Speech Support App, we conducted a language function assessment before and after the rehabilitation intervention. The standardized aphasia test (SLTA) and the SLTA supplementary test (SLTA-ST) (high-frequency words name recalling: 55 points; low-frequency words name recalling: 25 points), which are commonly used to evaluate aphasia in Japan, were used for evaluation. To quantify the results of the SLTA, we used the Z-scoring method (Akaboshi, 2007).

Higher cognitive functions (intellectual function, attention function and memory function) were assessed using the Raven's Colored Progressive Matrices (RCPM), the Trail Making Test–Japan, and the Rey–Osterrieth Complex Figure Test (ROCFT). In order to evaluate the effects of the Speech Support App on the patient's mental state, we evaluated the effects of motivation on the Speech Support App using the apathy scale and self-rated depression scale (SDS). In addition, a self-administered patient questionnaire and an evaluation of the application's effectiveness (usability, usefulness, attractiveness, and burden) were conducted.

### Functional magnetic resonance imaging evaluation methods

#### Image evaluation

We searched for differences in neural connectivity networks at rest and resting-state brain activity in language-related areas. In this study, the behavioral evaluation revealed the improvement of SLTA-ST, Trail Making Test Japan partA (TMT-J A), and Trail Making Test Japan partB(TMT-J B) in both patients. Based on these, we focused on the functional connectivity related to the language and attention networks. Signal changes in the brain regions related to the language and attention networks were extracted in a time series to analyze the brain networks related to this problem.

Rs-fMRI was performed using a 3-T MRI scanner (MAGNETOM Skyra, Siemens, Erlangen, Germany) equipped with a 32-channel head matrix coil. T1-weighted images were acquired using a 3D magnetization preparation-rapid acquisition gradient echo (MPRAGE) sequence (repetition time/echo time/inversion time = 2,300/2.96/900 ms; flip angle = 9 degrees; matrix size = 256 × 256; field-of-view for registration = 256 mm; slice thickness = 1.0 mm). Whole brain rs-fMRI scans were acquired with single-shot gradient echo-planar imaging (GE-EPI) (repetition time/echo time = 2,500/30 ms; flip angle = 80 degrees; resolution = 3 × 3 × 3 mm). Thirty-nine axial slices were acquired per volume, covering both cerebral hemispheres and the cerebellum. A total of 180 repetitions were acquired in approximately 7.5 min. During rs-fMRI, the participant was instructed to remain still and stare at the cross symbol on the monitor screen. Distortions were corrected using a field map calculated from the phase changes between the images at different echo times.

#### Rs-FMRI data preprocessing

The CONN software (https://web.conn-toolbox.org; Susan and Alfonso, 2012) was used for image data preprocessing, to correct for image misalignment due to head movement during imaging and imaging timing differences between slices, which was standardized to the Montreal Neurological Institute (MNI) coordinate system. Rs-fMRI images were preprocessed using the CONN-fMRI toolbox before analysis. The preprocessing steps included slice-timing correction; realignment; artifact detection; tool-based identification of outlier scans for scrubbing, segmentation, and normalization to MNI space; and spatial smoothing with an 8-mm full-width at half-maximum Gaussian kernel. Bandpass filtering was performed (0.008 < f < 0.09 Hz). The removed temporal confounders include the recalibrated parameter noise and Blood oxygenation level dependent (BOLD) signal from the white matter and cerebrospinal fluid. The distortion correction framework used was FUGUE (FMRIB's Utility for Geometrically Unwarping EPIs) in FSL.

To study the resting state language- and attention-related neural networks, we set up individual ROIs.

For the language networks, the following six ROIs were considered: the inferior frontal gyrus (IFG), insular capsule (IC), precentral gyrus (PreCG), supplementary motor area (SMA), supramarginal gyrus (SMG), and anterior/posterior superior temporal gyrus (STG).

The analysis of the attention networks was based on the results of the behavioral data on higher cognitive functions. For the attention networks, the following 10 ROIs were considered: the frontal pole (FP), superior frontal gyrus (SFG), middle frontal gyrus (MFG), inferior frontal gyrus (IFG), insular (IC), superior parietal lobe (SPL), supramarginal gyrus (SMG), thalamus, anterior cingulate gyrus (AC), and posterior cingulate gyrus (PC).

These ROIs were defined by the FSL Harvard-Oxford Atlas maximum likelihood cortical atlas (HarvardOxford-cort-maxprobe-thr25-1 mm.ni) implemented in CONN.

Correlation coefficients (bivariate) were calculated between the BOLD time-series signal of each ROI and the time-series signals of all other ROIs, and the Z-values after analysis were converted into correlation coefficients. In this study, Fisher's z-transform was performed on the Z-values between the ROIs related to language and attention from both patients. Since the objective of this study was to quantitatively evaluate the changes of functional connectivity related to the language and other cortical areas by intensive speech therapy, we mainly performed an ROI-based analysis. Moreover, regarding the seed showing main change by speech therapy, a voxel-based analysis was added to visually evaluate these changes.

## Results

**Figure 3:**
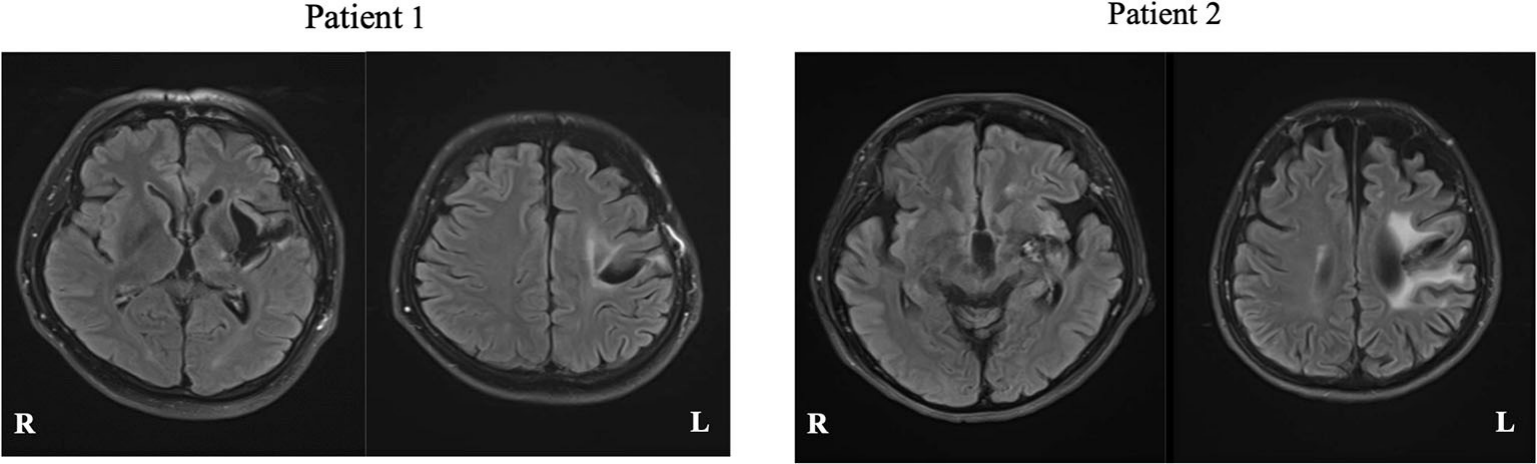
FLAIR magnetic resonance images of each patient. White matter lesions are seen, reflecting the history of cerebral hemorrhage.

Patient 1 was a 50-year-old, right-handed male (Edinburgh handedness assessment: 50 points), with 12 years of education (high school graduate), and a clinical diagnosis of hemorrhage in the left putamen. Neuroradiological findings were based on fluid-attenuated inversion recovery (FLAIR) imaging performed at Nagoya City University at 71 months after stroke onset, which showed a low signal area in the left putamen (Figure 3, left). Motor aphasia (Broca's aphasia) was observed in the evaluation of language function 71 months before rehabilitation intervention started.

Patient 2 was a 60-year-old, right-handed woman (Edinburgh handedness assessment: 80 points), with 15 years of education (university graduate), and a clinical diagnosis of left capsular hemorrhage. Neuroradiological findings were based on FLAIR imaging performed at Nagoya City University at 138 months after onset, which showed a low signal area in the left putamen (Figure 3, right). Motor aphasia (Broca's aphasia) was observed in the verbal function evaluation at 138 months performed before the rehabilitation intervention started.

### Evaluation before and after rehabilitation intervention

The results of evaluation before and after rehabilitation are listed in Table 1.

**Table 1:** Pre- and post-intervention results of language and attention function, higher cognitive functions assessments.

		**Patient 1**		**Patient 2**	
		**Pre**	**Post**	**Pre**	**Post**
Language assessment	SLTA (Z-score points)	60.85	60.78	59.49	60.62
	SLTA-ST (high- and low-frequency words)	High: 55 Low: 21	High: 54 Low: 25	High: 53 Low: 18	High: 54 Low: 21
Attention function assessment	TMT-J A (seconds)	130	34	121	49
	TMT-J B (seconds)	131	60	230	142
Higher cognitive functions assessment	RCPM (points)	34	31	32	33
	ROCFT (points)	11.5	12	14.5	19.5
Other assessments	SDS (points)	28	27	44	44
	Apathy scale (points)	5	0	16	14

SLTA, Standard Language Test of Aphasia; SLTA-ST, Supplementary tests for Standard Language Test of Aphasia; TMT-J, Trail Making Test Japan; RCPM, Raven's Colored Progressive Matrices; ROCFT, Rey–Osterrieth Complex Figure Test; SDS, Self-rating Depression Scale.

#### Aphasia assessment

In the SLTA-ST, both patients showed improvement in low-frequency word name recall after the rehabilitation intervention (Patient 1: 21 → 25 words; Patient 2: 18 → 21 words). The total SLTA score decreased slightly in Patient 1. In Patient 2, the SLTA scores improved. Since the Speech Support App is an intervention aimed at improving speech, in Patient 1, after the rehabilitation intervention, the “recitation of short sentences” score decreased, which affected the overall test scores; however, no significant change was observed in the other sub-items. In Patient 2, there was an improvement in “name recalling,” “recitation of short sentences,” “word enumeration,” and “reading aloud one kana character” scores.

#### Higher cognitive functions and attention function assessment

In the attention function test (TMT-J), both patients showed abnormal results that were far below the chronological average before the intervention; however, both TMT-J A and TMT-J B showed marked improvements after the rehabilitation. The RCPM, an intelligence test, showed a slight decrease in Patient 1 and a slight increase in Patient 2 after the rehabilitation intervention. However, the results of both patients were above average for their age group. The ROCFT, a visual memory test, showed improvement in both patients after the rehabilitation intervention. In particular, Patient 2 improved to the chronological average (Yamashita, 2007; Table 1).

#### Other evaluations

As shown in Table 1, there were no depressive symptoms or apathy after the rehabilitation intervention in either patient. In the self-administered questionnaire, questions related to feasibility, operability, usefulness, and attractiveness were all scored as highly satisfactory, and the respondents indicated that there were no psychological burdens or side-effects associated with the use of the Speech Support App. In the open-ended questionnaire, patients, family members, and medical professionals involved with the patients commented that the app increased the amount of speech in free-form conversations and words.

#### Functional magnetic resonance imaging evaluation

**Figure 4:**
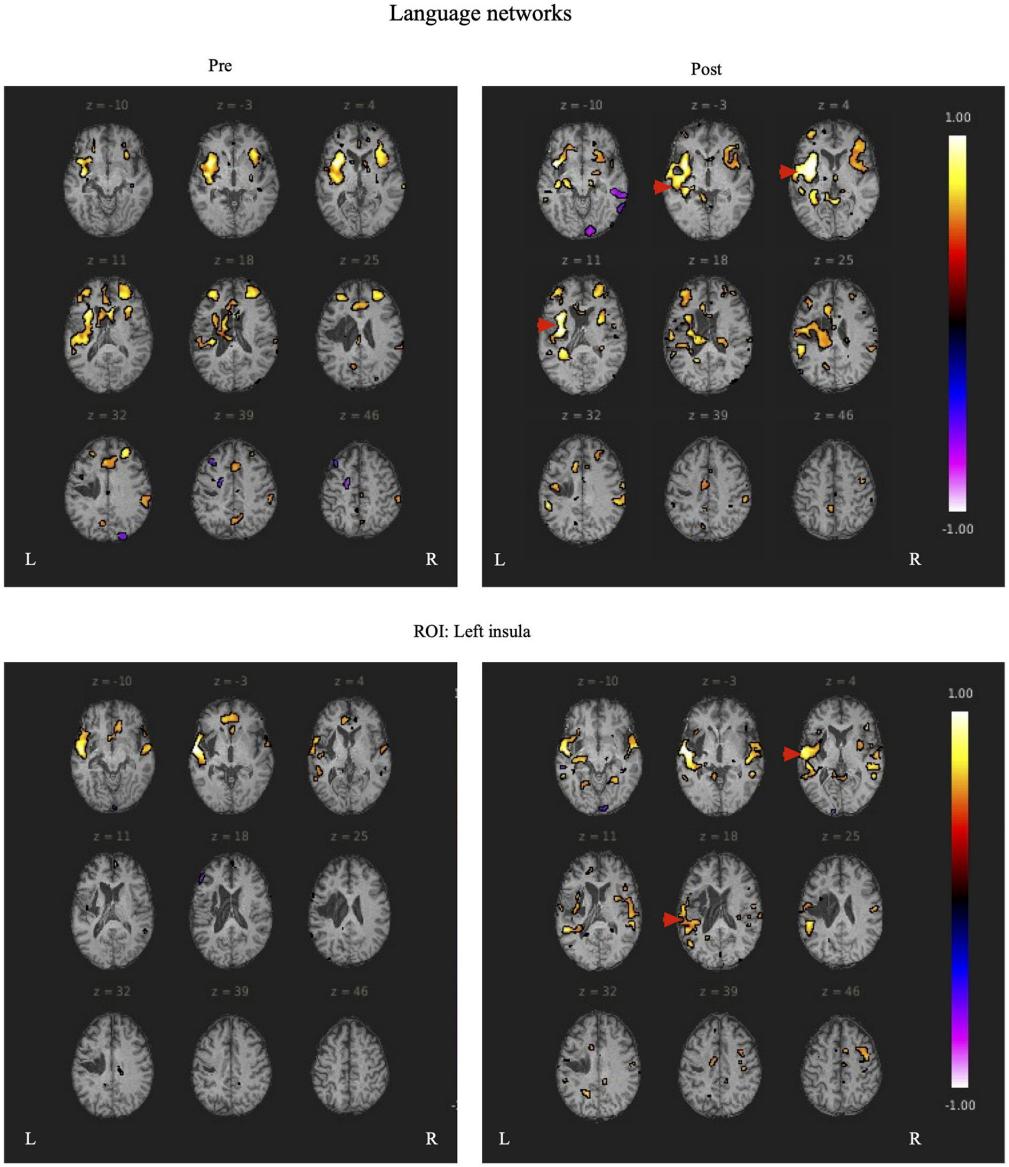
The left insular and left superior temporal gyrus are set as regions of interest (ROIs), and the results of Seed-to-Voxel are plotted. Correlation coefficients of more than 0.5 and < -0.5 in each ROI are shown in color. The left insular shows enhanced functional connectivity with the peripheral regions of the left insular, indicated by red arrows. In the left superior temporal gyrus, functional connectivity was enhanced in the temporal lobe region contralateral to the region around the left superior temporal gyrus, indicated by the red arrow.

**Figure 5:**
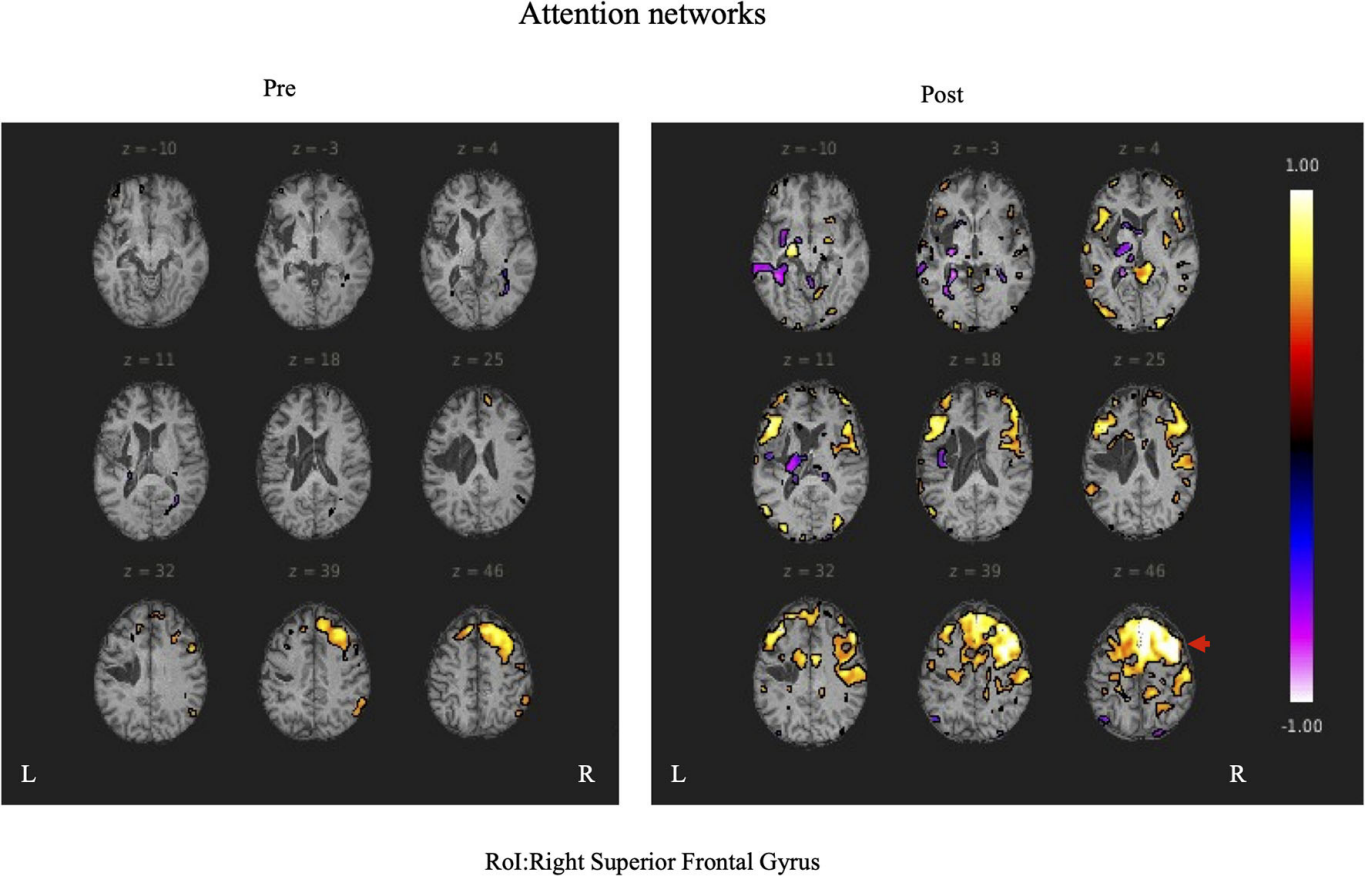
The right superior temporal gyrus was set as the region of interest (ROI) and the results of Seed-to-Voxel are shown. Correlation coefficients of 0.5 or more and −0.5 or less in each ROI are shown in color. The right frontal gyrus is indicated by red arrows.

During rs-fMRI imaging, although slight head movement were observed, related artifacts occur with movements of less than a few tenths of a millimeter. In this study, the patients had head movements of 2.0 mm or less, which were measured using the framewise displacement calculation of Jonathan et al. (2012). To confirm the changes in functional connectivity before and after the rehabilitation intervention, the top 10 ROIs with high increases in correlation coefficients are shown in Tables 2, 3. The following images show the results of the analyses on the left insular and left superior temporal gyri (Figure 4) and the right superior frontal gyrus (Figure 5), parts of the language and attention networks, respectively. Figures 4, 5 show regions of increased functional connectivity by rs-fMRI.

**Table 2:** Correlation coefficient results of region-of-interest (ROI) to ROI of language networks.

			**ROI to ROI**	**Correlation coefficient**	
				**Pre**	**Post**
Patient 1	Language networks	IC(L)	aSTG(L)	0.072	0.413
		IFGtri(L)	PreCG(L)	−0.130	0.253
			SMA(L)	−0.098	0.329
			networks.Language.IFG (L)	0.489	0.830
		IFG oper(L)	networks.Language.pSTG(R)	0.117	0.511
		PreCG(L)	pSTG(L)	−0.254	0.143
			networks.Language.IFG (L)	−0.028	0.320
		aSTG(L)	aSMG(L)	−0.172	0.155
			pSMG(L)	−0.191	0.262
		aSMG(L)	networks.Language.pSTG (L)	−0.049	0.300
Patient 2	Language networks	IC(L)	aSTG(R)	0.095	0.677
			aSTG(L)	0.013	0.461
		IFG oper(L)	IC(R)	−0.190	0.268
		PreCG(R)	pSTG(R)	−0.298	0.142
		pSMG(L)	IC(R)	−0.174	0.327
			aSMG(R)	0.085	0.514
		aSTG(L)	IC(L)	0.013	0.461
		pSMG(R)	aSTG(R)	−0.192	0.255
		SMA(L)	pSTG(R)	−0.114	0.384

R, right; L, left; ROI, region-of-interest; IC, insular cortex; IFG tri, inferior frontal gyrus pars triangularis; IFG oper, inferior frontal gyrus pars opercularis: PreCG, precentral gyrus; aSTG, superior temporal gyrus, anterior division; pSTG, superior temporal gyrus, posterior division; aSMG, supramarginal gyrus, anterior division; pSMG, supramarginal gyrus, posterior division; SMA, juxtapositional lobule cortex (formerly Supplementary Motor Cortex).

**Table 3:** Correlation coefficient results of region-of-interest (ROI) to ROI of attention networks.

			**ROI to ROI**	**Correlation coefficient**	
				**Pre**	**Post**
Patient 1	Attention networks	IC (R)	SPL (R)	0.029	0.371
			SFG (L)	−0.334	0.053
		SFG (R)	aSMG (R)	−0.086	0.324
			SMA (L)	0.135	0.514
		IFG tri (R)	SPL (R)	−0.052	0.322
			MidFG (L)	0.026	0.364
		IFG oper (R)	SPL (R)	0.136	0.505
		SPL (R)	Thalamus (R)	−0.105	0.245
			SFG (L)	0.048	0.389
		aSMG (R)	SFG (L)	−0.190	0.243
Patient 2	Attention networks	IC (R)	SFG (R)	−0.050	0.301
			MidFG (R)	−0.125	0.245
			IFG tri (R)	−0.027	0.379
		MidFG (R)	PC	0.212	0.603
		IFG tri (R)	Thalamus (R)	0.098	0.594
			AC	0.179	0.488
		IFG oper (R)	PC	−0.028	0.492
		aSMG (R)	pSMG (L)	0.085	0.514
		pSMG (R)	pSMG (L)	0.241	0.667
		Thalamus (R)	IFG oper (L)	−0.017	0.397

R, Right; L, Left; ROI, Region-of-Interest; IC, Insular Cortex; SFG, Superior frontal gyrus; MidFG, Middle frontal gyrus; IFG tri, Inferior frontal gyrus pars triangularis; IFG oper, Inferior frontal gyrus pars opercularis; SPL, Superior parietal lobule; aSMG, Supramarginal gyrus anterior division; pSMG, Supramarginal gyrus, posterior division; SMA, Juxtapositional lobule cortex (formerly Supplementary motor cortex; PC, Cingulate gyrus, posterior division; AC, Cingulate gyrus, anterior division.

In Patient 1, after the rehabilitation intervention, the language network showed functional connectivity enhancement in the left IC vs. anterior part of the left STG, left IFG (triangle) vs. left PreCG, left SMA, left IFG (language network), left IFG (opercularis) vs right STG (language network), left PreCG vs. posterior part of the left STG, left IFG (language network), anterior part of the left STG vs. anterior part of the left SMG, posterior part of the left SMG, anterior part of the left STG vs. left STG (language network).

In the attention networks, functional connectivity was enhanced in the right IC vs. right SPL, left SFG, right SFG vs. anterior part of the right SMG, left SMA, right IFG(triangle) vs. right SPL, left MidFG, left IFG (opercularis) vs. right SPL, right SPL vs. right Thalamus, left SFG, anterior part of the right SMG vs. left SFG.

In Patient 2, after rehabilitation intervention, the language network showed functional connectivity enhancement in the left IC vs. anterior part of the right STG and anterior part of the left STG, left IFG (opercularis) vs. right IC, right PCG vs. posterior part of the right STG, anterior part of the right STG vs. posterior part of the right SMG, posterior part of the left SMG vs. right IC and anterior part of the right SMG, left SMA vs. posterior part of the right STG. However, after the rehabilitation intervention, negative correlations were observed in some ROIs related to the language-related neural network in the left hemisphere.

In the attention networks, functional connectivity was enhanced in the right IC vs. right SFG, right MidFG, right IFG(triangle), right MidFG vs. PC, right IFG(triangle) vs right Thalamus, AC, right IFG (opercularis) vs. PC, anterior part of the right SMG vs. posterior part of the left SMG, posterior part of the right SMG vs. posterior part of the left SMG, right Thalamus vs. left IFG (opercularis).

## Discussion

This study showed that short-term intensive speech therapy using the Speech Support App improved pronunciation, improved word recall in everyday conversational situations, and attention in two patients with chronic aphasia. In addition, the rs-fMRI analysis showed increased functional connectivity of brain regions associated with language including left STG and left IC, and attention-related areas including right IC, SMG, AC, medial prefrontal cortex, and left SPL. These results show that even in chronic stroke, intensive speech therapy using this Speech Support App can improve language and attention functions related to the altered brain network.

### Relationship between aphasia and imaging assessment

Along with the progress of functional brain imaging, language research has progressed and the language cortex has been identified (Binder et al., 1997; Otsuki, 2018). The dual stream model of speech processing, in which the ventral pathway (mainly semantic processing) and the dorsal pathway (mainly phonological processing) are used for language information processing, has been proposed (Hickok and Poeppel, 2007; Fridriksson et al., 2016). The ventral pathway involves the primary auditory cortex to the STG and Broca's area, while the dorsal pathway involves the Broca's area to the SMG and Wernicke's area (Ishiai, 2017). Language processing is reported to occur through interaction between brain regions related to the dual stream model.

In both patients, SLTA-ST showed improvement (particularly in low-frequency words name recalling), while rs-fMRI also showed functional connectivity enhancement after rehabilitation, particularly between the left IC and left anterior STG; between the left IFG (triangular), left central PreCG, and left SMA; between the left central PreCG and left IFG (language network); and between the left STG and left SMG. These results showed that the left STG and the IFG (language network) were functionally connected and are consistent with the dorsal and ventral pathways of the dual pathway model; suggesting that the language network may have been reorganized or activated. Functional connectivity was enhanced between the left STG (anterior) and right IC; and between the left and right posterior STG.

Several studies revealed that in patients with Broca's aphasia, where the left hemisphere is destroyed, the right hemispheric region compensates for and contributes to language function (Gold and Kertesz, 2000; Abo et al., 2004; Winhuisen et al., 2005; Hartwigsen and Saur, 2019; Kiran et al., 2019). Therefore, it is possible that the improvement in language function in patient 2 was influenced by the strengthening of functional connections between the right hemisphere and the site of assumed language function. However, a longitudinal fMRI study of language function by Saur et al. (2006) showed that activation of auditory comprehension in the acute stroke phase failed to induce activation of the peri-lesional area, but activated the IFG and the adjacent IC, as well as the inferior hemisphere in the subacute phase, and activated the IFG cortex area in the left hemisphere in the chronic phase. The inferior frontal gyrus and adjacent insular gyrus were activated in the chronic stroke patient 2 evaluated in our study. These findings suggest that the improvement in language function in the chronic phase in patient 2 may not be due to the functional compensation of the right hemisphere, but rather to the enhancement of functional connectivity in another region of the left hemisphere.

For example, in a longitudinal study of aphasia using positron emission tomography (PET), de Boissezon et al. (2005) reported that all correlational analyses, both initial and follow-up PET, implicated the anterior part of the left inferior temporal gyrus, with a specific role for this region in lexical semantic processing. A PET study by Heiss et al. (1999) suggested that there is a hierarchical structure in the functional network associated with language during aphasia recovery. Thus, adequate recovery of language function occurs when the posterior left superior temporal gyrus is reintegrated into the functional language network of the Sylvian fissure within the left hemisphere. Good recovery of language has been reported in patients with preserved posterior left STG.

Furthermore, it has been suggested that the “ventral subcortical pathway,” which connects the posterior and superior temporal regions with the orbitofrontal cortex and dorsolateral prefrontal regions, is important in the semantic system, because semantic paraphasia appears in the naming task after electrical stimulation during surgery (Duffau et al., 2005). Wise et al. (1999) measured the brain activity of healthy patients using PET in a task in which participants had to repeat auditorily presented words. They found activity in the bilateral sensorimotor cortex, premotor cortex, basal ganglia, and upper cerebellum, as well as in the left anterior insular cortex, and concluded that the left anterior insular cortex is involved in speech motor planning.

In both patients in the present study, the IC and left STG were preserved, and functional connectivity was increased between both structures. This may be one of the factors that led to improvement of speech function and production, as the strengthening of functional connectivity between the two regions, which play a role in semantic planning and in speech movement, contributed to the improvement of aphasia. Furthermore, Richter et al. (2008) reported that the initial activation of the right IFG, IC, and other right hemispheric regions is positively correlated with the success of aphasia treatment, and that the activation of the right hemisphere before aphasia treatment indicates the possibility of language improvement in patients with chronic aphasia. Furthermore, the right hemispheres of both patients were originally more activated than the left, and the increased activation of the left hemisphere after the rehabilitation intervention may have had a positive effect on the improvement of language function.

In patients with motor impairment after stroke, increased functional connectivity correlates with improved motor function (Wang et al., 2010; Park et al., 2011; Chen and Schlaug, 2013; Liu et al., 2015). In addition, Gordon et al. (2020) reported the interaction between the Default Mode Network(DMN) and the language network. In the DMN subsystem, the frontotemporal subnetwork was specifically connected to the language network, and the dorsal subnetwork was specifically connected to the frontoparietal network. They concluded that specific circuits within the network structure of the brain serve as a series of connector hubs by allowing separate flows between the DMN and the frontoparietal and language networks. These suggest that the increased functional connectivity of the language network shown in this study may be somehow related to the improvement of language function.

Taken together, rehabilitation intervention using a speech therapy support application may contribute to the reorganization of the language network by strengthening functional connectivity and global brain activation in areas related to language function.

### Changes in attention functions and applications

Corbetta and Shulman (2002) reported the concept of attentional function in terms of dorsal (DAN) and ventral attentional networks (VAN). The DAN consists of the bilateral interparietal sulci and the frontal eye field, and is involved in the top-down processing of the attentional direction. The VAN consists of the right temporoparietal junction and the ventral frontal cortex, and is involved in the bottom-up processing of attentional reorientation. An rs-fMRI study conducted on Trail Maiking Test (TMT) performance also found an association between the “executive control network” and Trail Maiking Test part B(TMT-B) completion time scores. Faster TMT-B performance was reported to be associated with an increase in the functional connectivity (FC) between the bilateral interparietal sulci and the executive control network (Seeley et al., 2007). The executive control network coordinates sustained attention and working memory (Dorsolateral prefrontal cortex, lateral parietal cortex), response selection (dorsolateral prefrontal/frontal cortex), and response inhibition (ventral lateral prefrontal cortex). It also refers to executive functions such as planning, inhibition, updating, and attention allocation. In addition, resting state fMRI studies of TMT have reported that the FC between the bilateral superior parietal lobes and the prefrontal cortex is important for proper performance of TMT-B (Seeley et al., 2007; James et al., 2016). After the present rehabilitation intervention, functional connectivity was enhanced in prefrontal cortical areas, right IC, AC, medial prefrontal cortex, and SPL, which are related areas of DAN and executive control network. In the current study, we found changes in FC in brain regions similar to previous studies, and significant improvements in the TMT-J score, a test of attentional function. It is possible that the FC changes related to DAN and executive control network led to the improvement of attentional function. These results suggest that changes in the attentional neural network may be correlated with the test results. It is expected that the use of applications may also affect the transition of attentional functions. Mimura (2004) examined the brain infrastructure involved in the process of speech recovery and reported that poor performance in language functions showed no activation in the contralateral area of Broca's area in the right hemisphere. However, two of the patients in the study reported significant activation of the prefrontal cortex (Brodmann areas 9, 10, and 46) that was not seen in normal subjects, suggesting that it may reflect some load. In the present study, the two patients showed increased functional connectivity in brain regions related to language functions and activation in the right prefrontal region (particularly in the DAN), which was consistent with the report by Mimura (2004). Improvement of the DAN function is likely to be enhanced by intensively using the Speech Support App for a fixed period every day. In addition, because there are various stimuli in the Speech Support App, attention must be directed selectively to them, which induces a synergistic effect that naturally activates the attention function while training the language function. We speculated that this led to the improvement of not only language, but also attention, and it is possible that functional connectivity in attention-related brain regions was increased after using the Speech Support App, resulting in improved TMT-J performance.

In addition, Brownsett et al. (2014) reported that cognitive control is an important factor contributing to the likelihood of recovery in patients with chronic aphasia, and one of the key systems in cognitive control is thought to be the saliency network, involving the dorsal anterior cingulate cortex and adjacent cortices in the SFG, which is significantly correlated with communication. Both patients in our study had improved language function. This improvement in language function and communication ability suggests that the app may have induced an improvement in overall cognitive control function, which in turn affected the improvement in language function.

### Intervention using high-tech devices and changes in language function

When patients with Broca's aphasia underwent speech therapy using high-tech devices, their fluency improved, and they were able to produce a variety of words, and their expressive language doubled (Fridriksson et al., 2012, 2015). In addition, many chronic aphasic patients have shown improvement in language function in studies using high-tech devices (Ramsberger and Marie, 2007; Cassarly et al., 2021). The Speech Support Application developed in this study is likely to have a similar effect. Intensive speech and speech therapy in the chronic phase have been proposed to cause reactivation of language networks (Saur et al., 2006). Intensive speech therapy is also effective for patients with chronic aphasia more than 3 years after stroke onset (Code et al., 2010).

In summary, previous studies have suggested that short-term intensive speech therapy is effective in improving the language functions of patients. Similar results were obtained in intensive speech therapy using the app developed in this study, suggesting the usefulness of the Speech Support App.

### Research limits

Because of the small number of participants in this study (only two patients), careful consideration must be given to the interpretation of the effects of the Speech Support App and changes in the brain network.

A recent study involved 80 patients using already developed and evaluated application (Cassarly et al., 2021); while we only had two patients. However, unlike existing speech applications, this study developed a new speech support app that combines multiple sensory stimuli, such as, speech, text, and images, as well as speech movements. This study mainly evaluated the effectiveness of this newly developed application. In the future, we will conduct a comparative study with a larger population and a pre- and post-intervention study using a *post-hoc* test to confirm the effectiveness of the Speech Support App.

Moreover, as it has been reported in previous studies, resting-state functional connectivity varies across time and awareness states. Thus, we cannot totally rule out the effect of the patients' state of awareness on the changes in functional connectivity. In addition, to examine whether the degree of improvement in these aphasic patients was due to the Speech Support App or to natural recovery, comparisons should be made between patients using the application and those who do not.

## Conclusion

In this study, both patients who used the Speech Support App intensively for 8 weeks showed improvement in their language functions (particularly name recalling), and the patients, their families, and related medical personnel reported an increase in spontaneous speech and words. This indicates that using the Speech Support App may contribute to the improvement of language functions. The Speech Support App may contribute to behavioral changes in speech and cognitive functions in general by strengthening the functional connectivity of the language and attention networks and activating overall brain functions. In addition, the use of the Speech Support App did not result in negative mood or emotional changes, such as apathy. Moreover, it had the advantage of allowing users to engage in speech therapy by themselves without being restricted by time or place, making this a highly practical tool to support speech therapy in the future.

The difference between existing reports and the present patient is that it suggests that speech app for improving language function may be effective not only in improving language function but also in improving attention function. This is probably the first study to report that a speech app is involved in improving attention function. However, since the number of patients is still small and comparative studies have not yet been conducted, this point needs to be carefully examined. In the future, we will conduct longitudinal studies with larger numbers of patients, which we hope will continue the trends seen in the current study, and provide even stronger evidence for the usefulness of this new speech support app.

## Data availability statement

The original contributions presented in the study are included in the article/supplementary material, further inquiries can be directed to the corresponding authors.

## Ethics statement

The studies involving human participants were reviewed and approved by Nagoya City University Hospital Clinical Research Review Committee. The patients/participants provided their written informed consent to participate in this study. Written informed consent was obtained from the individual(s) for the publication of any potentially identifiable images or data included in this article.

## Author contributions

YU, HT, and NO contributed conception and design of the study. HT and KN contributed to the development of App. KI and HK contributed execution of the experiments. YK wrote the first draft of the manuscript. All authors contributed to manuscript revision, read, and approved the submitted version.

## Funding

This work was supported by KAKENHI, Grant-in-Aid for Scientific Research on Innovative Areas, Willdynamics (16H06403).

## Conflict of interest

The authors declare that the research was conducted in the absence of any commercial or financial relationships that could be construed as a potential conflict of interest.

## Publisher's note

All claims expressed in this article are solely those of the authors and do not necessarily represent those of their affiliated organizations, or those of the publisher, the editors and the reviewers. Any product that may be evaluated in this article, or claim that may be made by its manufacturer, is not guaranteed or endorsed by the publisher.
